# Introduction to carbon nanoarchitectonics for advanced applications in energy, environment and bio

**DOI:** 10.1039/d6na90009g

**Published:** 2026-02-17

**Authors:** Katsuhiko Ariga, Lok Kumar Shrestha, Qingmin Ji

**Affiliations:** a Research Center for Materials Nanoarchitectonics, National Institute for Materials Science (NIMS) 1-1 Namiki Tsukuba 305-0044 Japan ARIGA.Katsuhiko@nims.go.jp; b Graduate School of Frontier Sciences, The University of Tokyo 5-1-5 Kashiwa-no-ha Kashiwa 277-8561 Japan; c Department of Materials Science, Institute of Pure and Applied Sciences, University of Tsukuba 1-1, Tennodai Tsukuba 305-8573 Japan; d Herbert Gleiter Institute of Nanoscience, School of Materials Science and Engineering, Nanjing University of Science and Technology 200 Xiaolingwei Nanjing 210094 Jiangsu China

## Abstract

This editorial summarizes the history and outlines the importance of carbon nanoarchitectonics together with recent examples of energy, the environment, and bio-related applications.

## Abstract 2

Katsuhiko Ariga, Lok Kumar Shrestha and Qingmin Ji introduce the *Nanoscale Advances* themed issue on carbon nanoarchitectonics for advanced applications in energy, environment and bio.

## Graphical abstract

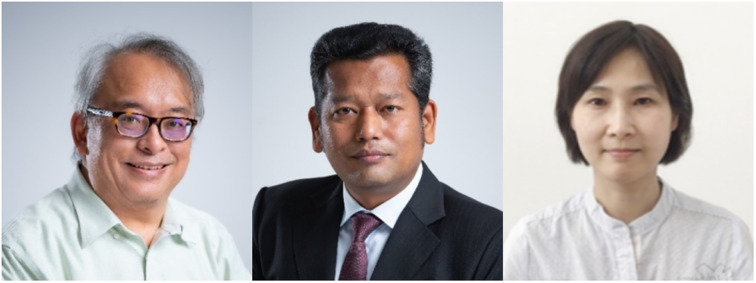

In this editorial, we will introduce the emerging field of ‘carbon nanoarchitectonics’. We will outline the significance of the terms ‘carbon’ and ‘nanoarchitectonics’ that comprise this concept. The importance of carbon is undeniable. It is an essential element for life as it forms the basic structure of all living organisms, including humans. It is therefore deeply connected to biological phenomena and social activities. A key example of this is carbon neutrality, which is vital in the fight against global warming. There is an urgent need to develop systems and functional materials that minimize carbon (carbon dioxide) emissions. Carbon materials possess a variety of nice properties, including being lightweight yet strong, having excellent electrical and thermal conductivity, being heat resistant, and being chemically stable. They are used in a wide range of applications, from aerospace to medicine. As well as being used as structural materials, they are also utilized in a variety of cutting-edge fields, including those requiring biocompatibility and for medical devices that utilize their X-ray transparency. In electronics, nanocarbon materials such as carbon nanotubes are being researched for use in semiconductor manufacturing processes, battery electrodes and next-generation display materials. Carbon materials are particularly useful as catalysts in the energy sector and there is high hope that they will become a substitute for expensive precious metals.

Carbon is an important element that is used in a variety of materials. It is therefore not surprising that it has been the subject of intensive research in recent years. Among these, nanocarbons have attracted considerable attention.^1,2^ Research into carbon materials with nanoscale structural dimensions, such as fullerenes, carbon nanotubes and graphene, is booming. The key point is that they are not just carbon materials, but also have a nanoscale structure. Indeed, it has been recognized that controlling the nanostructure, rather than just the material itself, is important for many materials other than carbon. A key concept for this in the 21st century is ‘nanoarchitectonics’.^3^ This term is briefly explained below.

We have acquired the science and technology to create a variety of functional materials for various application demands,^4–6^ primarily in the field of materials chemistry. Throughout our history, we have realized that controlling the precise structure of materials, as well as their properties, leads to improved functionality. Against this backdrop, the concept of nanotechnology emerged in the mid-20th century. Nanotechnology enables us to observe structures at the atomic and molecular level,^7,8^ manipulate them^9,10^ and elucidate the properties in nanospaces.^11,12^ By leveraging the power of materials chemistry to create materials and the impact of nanotechnology to control the nanoscale, our next goal is to develop a concept for the free creation of functional materials with controlled nanostructures. This is achieved through nanoarchitectonics, a post-nanotechnology concept that emerged in the early 21st century.^13^ It aims to combine the power of materials chemistry and nanotechnology to create materials with greater functionality, starting from basic units such as atoms, molecules and nanounits. In other words, nanoarchitectonics is a comprehensive materials development concept that integrates materials chemistry and nanotechnology. As all matter is composed of atoms and molecules, this methodology could be applicable to all materials. The ultimate goal of the nanoarchitectonics approaches would be a method for everything in materials science.^14,15^ In physics, the ultimate goal is a theory of everything, or a super unification theory. It would be great to have such a goal in chemistry too.

Thus, carbon nanoarchitectonics is the fusion of carbon, which is expected to have a wide range of applications, and nanoarchitectonics, which has the power to create many functional materials. *Nanoscale Advances* has published a themed collection titled ‘Carbon nanoarchitectonics for advanced applications in energy, environment and bio’ as a more advanced form of nanoscale chemistry. The papers collected there cover a wide range of topics, from the fundamental to the applied, and clearly demonstrate the significant impact of carbon nanoarchitectonics. Below is an overview of the papers published in this themed collection.

The mission of nanoarchitectonics is the creation of novel functional materials. For instance, ‘nanoarchitectonics’ surface synthesis has demonstrated remarkable capabilities in realizing desired carbon nanomaterials with atomic precision. Alcón *et al.*’s minireview, ‘Progress on quantum transport engineering in atomically precise anisotropic nanoporous graphene’ (https://doi.org/10.1039/D5NA00532A), summarizes progress in a specific type of nanoporous graphene constructed from two-dimensional (2D) arrays of graphene nanoribbons. This unique platform can be used to tune quantum electronic properties and 2D anisotropy. This methodology can be used for targeted applications at the nanoscale, down to the atomic and molecular levels. Observations of nanoscale material behaviour have also been reported. In their paper, ‘Electromigration-driven linear actuator operations of Co nanorods inside and outside multi-walled carbon nanotubes with stroke of tens of nanometers’ (https://doi.org/10.1039/D4NA00766B), Matsuyama and Kohno extrude solid cobalt (Co) nanorod fillers from multi-walled carbon nanotubes using electromigration, observing their behaviour *in situ* with a transmission electron microscope. For instance, reversing the direction of the electron flow causes the Co nanorods to be pulled into the host nanotube.

The publications also include research papers focusing on applications. Reflecting the suitability of carbon materials, applications in energy-related fields are particularly prominent. Gao *et al.*’s review paper, ‘Porous carbon-nanostructured electrocatalysts for zinc–air batteries: from materials design to applications’ (https://doi.org/10.1039/D4NA00847B), examines the oxygen reduction and evolution reactions in zinc–air batteries in detail through the use of advanced porous carbon materials. The paper integrates recent advances in porous carbon materials, providing crucial insights for developing next-generation, high-performance battery materials. In their report, ‘Terpyridine-functionalized single-walled carbon nanotubes towards selectivity in the oxygen reduction reaction’ (https://doi.org/10.1039/D5NA00281H), Sideri *et al.* demonstrate the stepwise chemical modification of single-walled carbon nanotubes with terpyridine ligands in the absence of metal, as well as in the presence of two different oxidation states of ruthenium (Ru). The comparative analysis revealed that the first coordination sphere of noble metals anchored on carbon nanomaterial lattices plays a crucial role in the thermodynamics and kinetics of the oxygen reduction reaction. This provides valuable insights into designing the nanostructure of efficient, carbon-based electrocatalysts. In their paper, ‘High-performance boron nitride/graphene oxide composites modified with sodium thiosulfate for energy storage applications’ (https://doi.org/10.1039/D4NA00937A), Shams *et al.* reported on the scalable synthesis of boron nitride/graphene oxide composites. This was achieved using a liquid-phase exfoliation method involving ultrasonic treatment. The composites were then prepared using sodium thiosulfate and exhibited excellent electrochemical properties, making them suitable for energy storage applications. Demonstrating high capacity, strong rate capability and outstanding coulombic efficiency, these composites are promising candidates for next-generation energy storage systems.

There have also been developments regarding environmental issues. Formaldehyde is a volatile organic compound that is a significant concern for the environment and human health. In their paper, ‘Functionalized carbon nanoparticles for smartphone-based sensing of formaldehyde’ (https://doi.org/10.1039/D5NA00865D), Cavallaro *et al.* reported on a fluorescent nanosensor for formaldehyde in both the aqueous and gas phases, which is based on dopamine-functionalized carbon nanoparticles. The sensitivity easily met the safety threshold recommended by the World Health Organization (WHO). Compatibility with smartphone-based detection could lead to the development of portable, low-cost devices for real-time monitoring. A review paper by Chakroborty *et al.*, ‘A review of emerging trends in nanomaterial-driven AI for biomedical applications’ (https://doi.org/10.1039/D5NA00032G), discusses the potential contribution to the medical field. Smart health tracking systems that integrate AI and nanoscience could represent a new frontier in overcoming various challenges. Targeted drug delivery, biosensing, imaging and other diagnostic and therapeutic fields can all benefit greatly from nanoscience in medicine. AI can assist in this area. For instance, the paper outlines the current challenges and potential opportunities in providing personalized healthcare using AI-assisted clinical decision support systems.

The papers in this themed collection reveal a wide range of developments. Furthermore, the concept of nanoarchitectonics is being employed in various ways that go beyond what is presented in these papers. For instance, applications range from controlling carbon skeletons to improving the catalytic function of oxygen reduction reactions,^16^ to localized assembly in biological systems that mimic the origin of life.^17^ Even this small collection of papers reveals a wide range of objectives. The concept of nanoarchitectonics is likely to have an impact on many areas of materials chemistry and nanoscale science. In order to realize its enormous potential, it has been proposed that AI technology be incorporated into nanoarchitectonics.^18^ In any case, functionalizing and applying carbon materials are essential to meet societal demands. This themed collection provides a glimpse of this and may serve as an indication of the future of materials and nanoscale sciences.
